# Perceived Risk of Job Instability and Unethical Organizational Behaviour Amid the COVID-19 Pandemic: The Role of Family Financial Pressure and Distributive Injustice in the Tourism Industry

**DOI:** 10.3390/ijerph19052886

**Published:** 2022-03-02

**Authors:** Ibrahim A. Elshaer, Alaa M. S. Azazz, Samy Wageh Mahmoud, Marwa Ghanem

**Affiliations:** 1 Department of Management, School of Business, King Faisal University, Al-Ahsaa 380, Saudi Arabia; 2 Hotel Studies Department, Faculty of Tourism and Hotels, Suez Canal University, Ismailia 41522, Egypt; samy_wageh@tourism.suez.edu.eg; 3 Department of Tourism and Hospitality, Arts College, King Faisal University, Al-Ahsaa 380, Saudi Arabia; marwamagdy00@gmail.com; 4 Tourism Studies Department, Faculty of Tourism and Hotels, Suez Canal University, Ismailia 41522, Egypt

**Keywords:** job instability, UOB, distributive injustice, financial pressure, COVID-19

## Abstract

The economic disaster precipitated by the pandemic of COVID-19 changed people’s perceptions of ordinary job stability and elevated it to an ultimate high level. To avoid being laid off, employees who are concerned about job stability may engage in unethical activities in the name of their employer. In this study, the influence of job instability on unethical organizational behaviour (UOB) was investigated through the mediating role of family financial pressure and distributive injustice. Perceptions of 830 employees working in hotels (5-star and 4-star) and travel agencies (Category A) were explored and further analyzed using structural equation modelling. The results asserted that family financial pressure and distributive injustice partially mediated the effects of job insecurity on UOB. Important insights on theoretical and practical implications were further deliberated towards the end of this study.

## 1. Introduction

Since the world’s COVID-19 outbreak, the hospitality and tourism industries have suffered a serious downturn [1,2,3,4,5,6]. Organizational dynamics have changed as a result and organizations faced with the need to drastically reduce activities [5,7], jobs and working hours, or lay off employees [8]. As a result, productivity and organizational competitiveness have generally decreased [9]. Workers in different industries have experienced not only their workplaces being jeopardized, but also their career prospects become insecure and unstable [10]. The theory of insecurity of jobs defined job insecurity as “perceive powerlessness to maintain desired continuity in a threatened job situation” [11]. It argues that when people believe they have no authority to defend themselves against employment threats, they feel insecure. While job insecurity is an employee’s subjective perception, job instability is caused by external objective factors such as political instability [8], a shrinking workforce, or the threat of future job loss [12]. Work instability has similar consequences to job insecurity and is a major source of economic stress for employees. It has a substantial impact on their well-being, competitiveness, and mental health [13]. Job instability could be attributable to the employee in question, with some just unable to cope with the stress caused by job demands or a high level of control [14]. Additionally, job instability is caused by labour market turmoil [15], in which economic actors have reengineered the business sector to survive and maintain competitiveness and have regularly suffered from increased competition within the industry [16], along with social, economic, and/or health crises [17].

During the COVID-19 pandemic, the fear of employment instability produced substantial physical and mental health problems. Researchers [3] found that job instability had a negative impact on employees’ organizational behaviour, such as increased unethical organization activity in the name of the company [3]. Employees were found to engage in unethical organizational behaviour (UOB) to benefit their organizations [18]. Their attempts may aid organizations with short-term gains that could be harmful to the organizational image in the long run [19]. Exaggerating the benefits or value-added of a firm’s products or services to consumers, for example, may reduce customer loyalty in the long run while providing short-term benefits to the company.

Organizational unethical behaviour benefits the organization rather than the employee. For instance, providing inaccurate information to a client in order to meet the corporation’s quarterly established targets might be one of these activities. Additionally, when employees’ moral resources are depleted, their cognitive powers are reduced, and their subsequent ability to self-regulate is harmed. As a result, employees may choose to engage in unethical activity that benefits the business or themselves.

Furthermore, as the perceived risk of job instability rises, so does family financial pressure, and as a result, employees may engage in UOB in order to decrease such stress (i.e., if employees conduct UOB, they may acquire advantageous outputs—a decrease in family financial pressures) [20].

Perceived risk of employment instability, according to stress-related theories, threaten essential resources and conjure uncertainty, and hence is regarded as stressful and strain-inducing [21,22]. Job instability, on the other hand, is portrayed as a psychological contract breach for permanent workers [23] or as an imbalance in effort and reward in social-exchange-related theories (feeling job unstable lessens rewards that employees obtain from their loyalty and investments) [24]. As a result, feeling job instability lowers justice perceptions, which might drive people to act unethically to save their jobs [3]. This study aims to explore the relationship between the perceived risk of job instability and employees’ UOB. This is tested in the context of growing fear of employment loss that would combine with the tendency to engage in UOB that may provide short-term organization benefits. In addition, the current study examines the mediating influence of family financial pressure and distributive injustice in the relationship between job instability and UOB. Although the study of job instability has received much attention, still, further research is needed to better understand how employees adapt to and manage it [25,26]. Therefore, this study offered a model that could help academics and practitioners better understand how job instability may influence employees’ UOB via the mediation role of family financial pressure and injustice distribution. This model can also have some implications for practitioners. Employees that engage in unethical behaviour in the name of their company may be doing it for personal gain, and their actions may harm the company’s reputation. As a result, managers should abstain from sending signals that cause employees to fear job instability in the workplace during a pandemic.

## 2. Theoretical Background and Hypotheses Development

### 2.1. Perceived Risk of Job Instability and Unethical Behaviour

The topic of risk perception has long been a focus of research in a variety of fields [3,27,28,29]. In the hospitality and tourism industry, particularly, perceived risk and its effect on human behaviours have been widely researched [3,4,28,30]. The subjective assessment of the likelihood of undesirable events is referred to as perceived risk [28]. This concept implies one’s perceptions of prospective risks or the chance of a loss [28]. A person’s perception of risk is based on both physical and mental factors (e.g., sickness, death, stress, job instability) [28,31]. As suggested by [30], in the tourism industry, the primary concern of individuals is the perceived danger of employment instability. Fear, anxiety, uneasiness, and discomfort among employees are all components of the perceived risk that is the source of this concern [28,29]. Consequently, in the current research, the term “perceived risk” refers to a person’s anxiety about job loss and employment instability as a result of working during the COVID-19 pandemic [32]. In a pandemic era, employees in the hospitality industry confront the impending risks of infection and employment instability [33].

**Figure 1:**
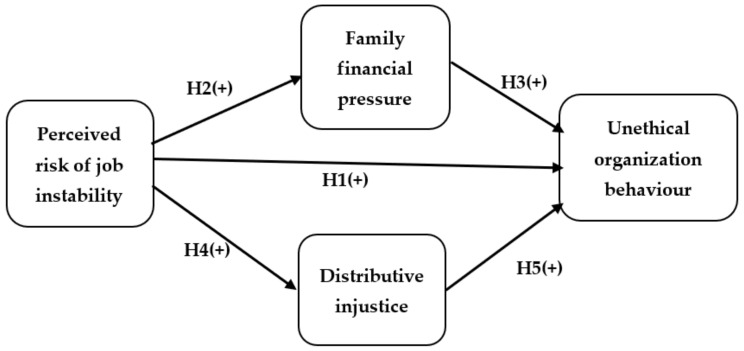
Research framework and hypotheses.

Job instability has negative effects on employees’ behaviour, such as diminished intrinsic motivation, unethical behaviour, and greater turnover intentions [3]. Many employees are afraid of viral infection because the COVID-19 pandemic has a general high infection and death rate, which can lead to negative psychological problems such as depression and anxiety [34]. Similarly, in today’s tourism business, hospitality employees encountered uncertainty and the fear of becoming infected with a virus and losing their jobs is greater than ever. As a result, the decision-making processes and behaviours of employees are influenced significantly by risk perception of job instability [3,27,29]. This perceived risk of job instability is similarly vital in clarifying the procedure of producing employees’ favourable or unfavourable reactions and behaviours [32,35]. Prior empirical studies have revealed that workers may behave unethically to promote self-interest [36,37], the group [38], their family ([39], or in the name of the organization [18,40]. Unethical behaviour might contain following practices: (1) obstruct others’ abilities to improve employees’ personal relationships, reputations, and job success [41]; (2) employees’ voluntary practices and behaviours contradict the organization standards and values and in so doing threaten the organization image [42]; and (3) out of an offender’s envy, pursue retaliation against a colleague [43]. Accordingly, the following hypothesis is suggested as shown in Figure 1:

**Hypothesis** **1** **(H1).** *Employee perceived risk of job instability is positively correlated with UOB*.

### 2.2. Perceived Risk of Job Instability, Family Financial Pressure, and Unethical Behaviour

Employees and their families struggle to manage their finances because of the high rate of job instability [44]. Despite the significant increases in recent years in our knowledge of the influence of job instability on health, stress, and well-being [45,46], causality is hard to be presumed. Financial pressures are likely to make job instability more painful, which in turn provokes financial pressures [47,48]. A limited number of studies have examined the link between job instability and employees’ financial well-being and related financial pressures. The impacts found to range from significant [48] to nonsignificant [47]. As a result, the interaction between job instability and financial pressure may require more research.

Furthermore, family financial stress affects not only impoverished people struggling to save for necessities but also more opulent people attempting to keep up with their peers. When facing severe financial pressures from family members, an employee’s primary goal would be to alleviate those pressures. The greater the urgency, the more important this goal becomes. As a result, when employees face significant family financial issues, they are more inclined to concentrate on receiving financial benefits from their organization [49]. Employees may place a high value on unethical practices in such situations because it not only reduces their personal annoyance and tension but also improves the quality of their family’s financial issues. Many types of unethical behaviours in the name of the family—such as taking organization resources home for usage or bringing family members to the workplace to benefit from the organizational possessions—are closely connected to financial advantages that can help relieve family financial pressures. In other words, when family financial pressure is high, employees may become more likely to participate in unethical behaviours. This suggests the following hypotheses:

**Hypothesis** **2** **(H2).** *Employee perceived risk of job instability is positively correlated with family financial pressure*.

**Hypothesis** **3** **(H3).** *Family financial pressure is positively correlated with unethical behaviour*.

### 2.3. Perceived Risk of Job Instability, Distributive Injustice, and Unethical Behaviour

Job instability might provide the impression that the psychological contract between employees and their company has been broken. The psychological contract is a concept introduced by equity [50] and organization justice theories [51]. Both are based on social exchange theories [52]; they highlighted how workplace motivation and involvement are impacted by the perception of the employee–organization interrelationships and by the directions that rule it. According to Adam’s equity theory, employees’ attitudes and behaviours in the workplace are based on their assessment of equity between inputs and outputs obtained by the business, as compared to other coworkers or their own goals and representations. Organizational fairness theory has advanced Adam’s equity theory by broadening the definition of equity to include more than just outcome distributions and allocations (distributive justice), but also the adequacy and fairness of the processes employed to regulate outcome distributions (procedural justice). Later, Bies and Moag [53] also presented the significance of interpersonal treatment that people obtain when procedures are employed; they described these practices as “interactional justice”.

As revealed by [54], when employees are confronted with uncertainty (i.e., perceived risk of job instability), they tend to apply justice judgements more frequently and justice consequences become greater in the existence of several causes of uncertainty. Fair treatment will serve as a guide in this scenario, directing personal attitudes and behaviours in the face of uncertainty. Employees will be able to sustain positive behaviour and attitudes for the company in this manner. As a result, based on fairness heuristic theory and uncertainty management theory, when people encounter job instability, they rely on their perceptions of fair or unfair treatment to choose how to respond. If they are unsure about their justice decisions, they use cognitive shortcuts to address the problem, such as replacing one sort of justice for another. When employees have access to justice information and believe they have been treated fairly, they will demonstrate the good consequences of justice in terms of positive behaviour and actions toward the organization. On the other hand, if employees think that they have been unfairly treated (i.e., distributive injustice), they will participate in self-protective behaviours or even in unethical behaviours to reduce uncertainty [54]. Thus, the following hypotheses are proposed:

**Hypothesis** **4** **(H4).** *Employee perceived risk of job instability is positively correlated with distributive injustice*.

**Hypothesis** **5** **(H5).** *Distributive injustice is positively correlated with unethical behaviour*.

## 3. Methodology

### 3.1. Instrument Measurement

Scales of this study were employed following a study of existing theoretical items and an extensive literature review. Accordingly, four aspects were identified, each with a set of items tailored for the tourism sector. Table 1 illustrates the operationalization of the main concepts of this study. The items measuring the perceived risk of job instability were constructed using the multi-item scale proposed by [35]. A sample item is “I have an unstable job environment”. Family financial pressures were measured by three items based on the study of [55]. A sample item is “My family has difficulty paying its monthly bills”. Colquitt’s four items of distributive justice [56] were revised to measure distributive injustice; a sample item is “I feel that the outcome process does not reflect the effort I have put into my work”. Finally, UOB was measured by seven items derived from [18], a sample item is “If it would benefit my organization, I would withhold negative information about my company or its products from customers and clients”.

A five-point Likert scale was used with a starting point of 1 (strongly disagree) and a maximum of 5 (strongly agree). The study’s pilot instrument was examined by a small group of employees (10) and academics (4). It was read and clarified for readability, applicability, and comprehension. The questionnaire made it clear that confidentiality and anonymity would be protected in the pursuit of reliable and consistent data. Owing to the study’s use of a self-report questionnaire, common method variance (CMV) could be an issue [57]. To prevent any potential CMV concerns, a single-factor analysis of Harman was utilized, with the numbers of recovered components limited to one in the SPSS exploratory factor analysis (EFA) assessment without rotation. As a result, CMV is not a cause for concern, as just one component was identified that explained 35% of the variance.

### 3.2. Participants and Data Collection

The study population consists of all full-time nonmanagerial employees at hotels (5-star and 4-star) and travel agents classified as category A. Lacking an accurate published database of Egyptian tourism employee numbers, hiring and training 25 enumerators from university students was the most suitable method to obtain data from the targeted population in Cairo (Egypt’s capital and most populous metropolitan region). This technique was used to circumvent the limited response rates associated with online data collection and/or traditional mail methods [58,59,60]. Data collection was conducted following COVID-19 public health precautions. Before participating in this study, respondents were requested to sign a consent form. Enumerators were instructed to read the questionnaire clearly and to complete responses. The hired and trained enumerators were capable of randomly contacting four to five employees (to avoid over or underestimation) in 120 hotels and 110 category A travel agents. A total of 1000 employees were contacted and 100 refused to participate; therefore, enumerators read and completed 900 surveys. Seventy were discarded due to insufficient responses, leaving 830 questionnaires suitable for study. The data collection period lasted three weeks in May 2021. The sample size of 830 employees was sufficient for structural equation modelling (SEM) [61].

Our sample size of 830 was sufficient for SEM testing since it complied with Nunnally’s [62] criterion of at least ten respondents per item. Because our scale has 19 indications, our sample size surpasses the needed 190. It satisfies Boomsma’s [63] sample size criterion based on the ratio of indicators (*p*) to latent unobserved variables (k), which is 4.75 (19 observed indicators/four latent constructs) in our paper, necessitating a minimum sample size of 200; and it complies with the requirement indicated by Hair et al. [61] for a minimum sample size of 100 to 150 to obtain acceptable maximum likelihood estimation (MLE) solutions. Additionally, whereas Krejcie and Morgan [64] recommend a sample size of 384 when the population surpasses 1,000,000, our study used a sample size of 830, surpassing the guidelines. The primary benefit of such a high number is that it enables the application of advanced data analysis techniques such as SEM. It facilitated the successful investigation of the research variables’ interdependence assumptions in this study.

## 4. Data Analysis Results

### 4.1. Descriptive Statistics

A total of 120 (52%) hotels (five-stars) and 110 (48%) travel agents (category A) contributed to this study. The study’s 830 participants were full-time employees, 65% were male and 35% were female, the majority (75%) were married, and 80% were between the ages of 25 and 44. The majority of those surveyed had earned a bachelor’s degree (72%). In terms of tenure, 600 respondents (almost 73%) had more than five years’ experience with their companies, while the remainder had been with the organization for less than five years (see Table 2). Their replies varied from 5 to 1, with 5 indicating “strongly agree” and 1 indicating “strongly disagree”. The mean values varied between 3.54 and 4.14, whereas the standard deviation values ranged between 668 and 1.328, suggesting that data were more dispersed and less concentrated around the mean [65]. The skewness and kurtosis readings (distribution of scores) indicated that there were no values more than +2 or less than −2, suggesting a normal univariate distribution [66], as shown in Table 2.

### 4.2. Confirmatory Factor Analysis (CFA)

To examine the scale’s convergent and discriminant validities, a first-order CFA test was done using the maximum likelihood (ML) estimate technique. Four dimensions (perceived risk of job instability, family financial pressure, distributive injustice, and unethical organization behaviour) were exposed to CFA with their associated variables. To analyze the fit of structural and measurement models, many goodness-of-fit (GoF) criteria were utilized, as proposed by [61,67,68], including: “normed chi-square” (chi-square divided by degree of freedom), “Tucker–Lewis index” (TLI), “comparative fit index” (CFI), “standardized root mean squared” (SRMR), and “root mean square error approximation” (RMSEA), and “parsimony comparative fit” (PNFI). The findings of the CFA’s GoF analysis indicated that the fit of the model was satisfactory (see Table 3). Cronbach’s alpha values and composite reliability were employed to verify construct reliability (CR). The study’s four dimensions had the following CR scores, as shown in Table 3: perceived risk of job instability (0.965), family financial pressure (0.947), distributive injustice (0.972), and UOB (0.978). All of the results were more than the stipulated cutoff value of 0.70, suggesting a high degree of internal consistency [67].

Additionally, the convergent validity of the measuring scale was established because all variables had relatively high and significant factor loadings (FL) (Table 3). As demonstrated in Table 3, the FL values varied from 0.803 to 0.984, above the suggested threshold of 0.50 [61]. Additionally, the values for the “average variance extracted” (AVE) for all research dimensions (perceived risk of job instability, family financial pressure, distributive injustice, and unethical organization behaviour) were 0.827, 0.856, 0.899, and 0.863, respectively (Table 3). All AVE values were more than 0.50, indicating satisfactory convergent validity [61]. The values of AVE were also found to be higher than all of the “maximum shared variance” (MSV) scores (Table 3), suggesting that the discriminant validity was good [61]. The square root of the values of AVE for every dimension was greater than the intercorrelation values among dimensions, indicating that the research measurement had good discriminant validity [61,67,68] (Table 3).

### 4.3. Structural Model Results

**Figure 2:**
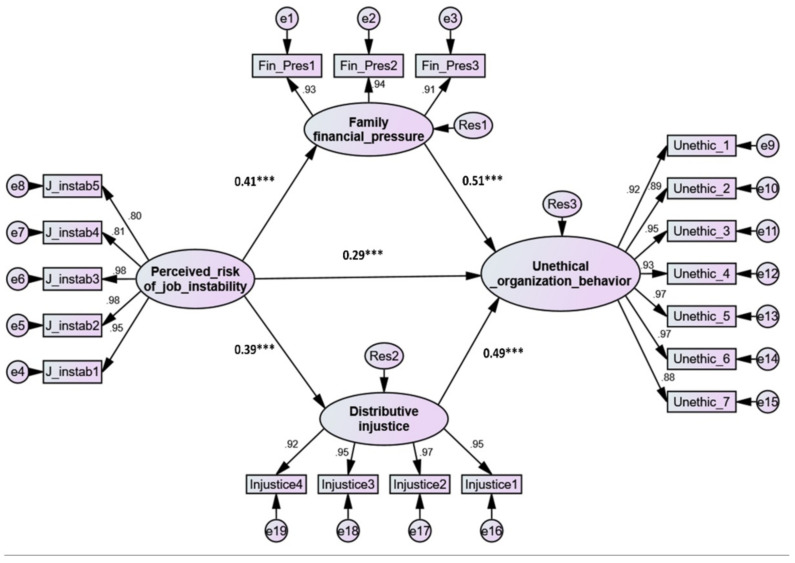
The tested structural and measurement model. *** significance <0.001.

The current study used a confirmatory technique. First, a thorough review of the literature helped in developing the theoretical conceptual model, and afterwards, primary data were acquired to check whether it matched [66]. The theoretical (structural) model was thus either supported or rejected depending on its ability to satisfy a model fit criterion. As seen in Table 4, the structural model fit the observed data correctly: χ^2^ (147, *n* = 830) = 591.07, *p* < 0.001, normed χ^2^ = 4.021, RMSEA = 0.022, SRMR = 0321, CFI = 0.916, TLI = 0.986, NFI = 0.912, PCFI = 0.701, and PNFI = 0.698. The study hypotheses were evaluated after attaining a satisfactory model fit. Each relation included in Figure 2 illustrates a research hypothesis.

Five hypotheses were proposed in this study. The first hypothesis, which examined the influence of perceived job instability on UOB (H1), is supported (*t*-value = 5.421, *p* < 001) with a significant path coefficient of 0.29, indicating a positive direct association between the two variables. Similarly, the SEM results indicated that the perceived risk of job instability has a significant positive influence on family financial strain (H2) (*t*-value = 9.986, *p* < 0.001) with a strong path coefficient of 0.41, hence confirming the second hypothesis (H2). Additionally, hypothesis three examined the effect of family financial pressure on UOB; the SEM results revealed a positive and significant (*t*-value = 13.645, *p* < 0.001) relationship between the two dimensions with a path coefficient of 0.51, thereby asserting the third hypothesis (H3). Moreover, the effect of the perceived risk of job instability on distributive injustice was significantly positive (*t*-value = 7.789, *p* < 0.001) with a path coefficient of 0.39, thus confirming the fourth hypothesis (H4). Finally, the effect of distributive injustice on UOB was also shown to be positive and significant (*t*-value = 7.789, *p* < 0.001, path coefficient = 0.49); therefore, the fifth hypothesis was supported.

To examine the mediation role of family financial pressure and distributive injustice in the link between perceived job instability and UOB [69,70], recommendations were applied. Zhao et al. [70] argued that for direct-only nonmediation effects, only direct path coefficients should appear with a significant *p*-value; for complementary mediation, both indirect and direct relationships should appear with a significant *p*-value and the same sign exist. Finally, competitive mediation is established when the direct and indirect effects are statistically significant and have an opposing sign. As seen in Figure 2, all regressions are significant with the same sign, as shown in Table 4. More precisely, the direct path from perceived risk of job instability to UOB is positive significant (β = 0.29, *t*-value = 5.421, *p* < 0.001), and perceived risk of job instability significantly, positively, and directly affect family financial pressure (β = 0.41, *t*-value = 9.986, *p* < 0.001) and distributive injustice (β = 0.39, *t*-value = 7.879, *p* < 0.001). In return, family financial pressure had a positive, direct significant effect on UOB (β = 0.51, *t*-value = 13.645, *p* < 0.001). Likewise distributive injustice had a positive, direct significant effect on UOB (β = 0.49, *t*-value = 12.601, *p* < 0.001). These findings confirm the complementary mediation of family financial pressure and distributive injustice in the relationship between perceived risk of job instability and UOB standardized indirect and total effects may also be analyzed in the SEM result to identify mediation effects [69]. The standardized indirect impacts from the perceived risk of job instability to UOB through the mediating role of family financial pressure and distributive injustice raises the direct impact from (β = 0.29, *p* < 0.001) to an equal effect (β = 0.54, *p* < 0.001). This implies that UOB increased by 25% through the mediating role of family financial pressure and distributive injustice. In addition, the structural model demonstrated a high degree of explanatory power (R2), accounting for 60% of the variance in UOB (Table 4).

## 5. Discussion and Implications

Many businesses, including the hospitality and tourism sectors, have been adversely affected by the COVID-19 outbreak. Lockdowns, home quarantine, social distancing, and other travel restrictions have all been working to increase the COVID-19 effects, forcing many hospitality firms to temporarily close [7] or lay off many of their employees. The lack of proper employment protection legislation or economic conditions, especially in underdeveloped nations (e.g., Egypt), may subject employees to job instability [71,72]. This study examined the impact of perceived risk of job instability on UOB among hospitality and tourism personnel in a developing nation (Egypt) during the pandemic of COVID-19. It investigated the psychological mechanism that would drive some employees with job instability to engage in unethical activities and decisions in their organizations. Family financial pressure and distributive injustice were examined as mediating dimensions. A total of 830 employees in the hospitality and tourism sectors were surveyed. Perceived risk of job instability was shown to raise family financial pressure, perpetuate distributive injustice between employees, and promote unethical organizational practices. These findings supported predictions and earlier results of the few studies that examined these correlations in non-Western nations [3,17,18,19]. However, little research examining these correlations has been undertaken in non-Western nations [3,73]. As a result, our research added to the body of knowledge by examining these interactions in the Egyptian setting.

Employees can overcome their sense of job instability by working hard and requesting assistance from others [8,71]. However, there is scarce research that examines employees’ reactions to job insecurity by practicing behaviours that are unethical but are in the name of the company. Job instability was demonstrated to directly develop unethical actions of employees in the name of their companies. Employees may undertake UOB that would, in turn, aid them to be viewed as useful to their companies and, thus, keep employment or employment advantages. These findings complement the results of [3,4,5,18,19] who argued that employment instability increases employees’ unethical practices. Moreover, according to [49], the perceived risk of employment instability has a significant influence on family financial strain, particularly during the COVID-19 pandemic. When workers feel a possible job loss and are insecure, they thus begin to rethink their position and future career path within the organization [73]. This, in turn, causes many of them to behave unethically and seize financial benefits from their organization, such as taking organizational resources home for personal use or bringing one of the family members to the office to benefit from organizational possessions—these actions are inextricably linked to financial benefits that can alleviate family financial strain. In addition, the current study shows that the perceived risk of employment instability increases distributive injustice. This finding is in line with [54] who argued that work insecurity is linked to job discontent and distributive injustice [54]. In return, employees may act unethically in the name of the corporation, hoping that this would be rewarded with continued employment.

This study has several other implications for both practitioners and academics, noted as follows. The perceived risk of employment instability should be a top concern for senior management in hospitality and tourism firms, especially during crises, since it has been evident to have several negative effects on workers and the organization. Employees’ perceived risk of job instability increases strains and financial pressures and expands perceptions of workplace inequity and distributive injustice, which, in turn, raise unethical behaviours in organizations. This would eventually decrease organizational effectiveness. Moreover, this study proved the mediating role of family financial strain and distributive injustice in strengthening the effect of perceived risk of job instability on UOB. Examining these correlations may help academics gain a better grasp of the nature of the links between job instability and unethical behaviours in organizations.

Additionally, the results of this study have particular implications for hospitality and tourism sector management. In developing nations (e.g., Egypt), where organizations may suffer extra economic consequences during crises and where unemployment is generally significantly high [26], perceived risk of job instability would have a detrimental effect on hospitality and tourism enterprises. Employees’ feeling of job instability may jeopardize the hospitality industry’s reputation and goodwill if followed by unethical activities in the name of the firm to keep employment or some financial privileges. Therefore, hospitality and tourism business managers should avoid conveying signals to their staff that they are at risk of losing their employment, especially during crises when employee solidarity is needed for the survival of the business. Thus, it is crucial that managers recognize that confusion or misinterpretation of management practices can contribute to employees’ sense of instability, which increases their financial stress and workplace disparity, distributive unfairness, and results in unethical behaviour that would risk business reputation and effectiveness.

## 6. Limitations and Areas for Further Research

This study contains five limitations and several avenues for further research. First, the results revealed that family financial pressure and distributive injustice partially mediated the relationship of job instability and unethical behaviours in organizations. For further insights, future studies can examine several other variables (e.g., social loafing, trust in supervisor, compensations, and job satisfaction) that may have the ability to mediate perceived risk of job instability and UOB. Second, for a more comprehensive approach, further studies can examine not only the antecedents of UOB but the consequences and possible impacts on organizational image and profitability. Third, because the data are cross-sectional in nature, causal relationships between variables cannot be strictly drawn. Fourth, while we aimed to prevent CMV following [74] suggestions, future researchers may use longitudinal data or a combination of data sources to test the proposed model of the study. Fifth, by utilizing a multigroup analysis method, the proposed model may be used to examine these correlations in diverse context (industry or country) [75].

## 7. Conclusions

The aim of this study was to investigate the impact of the perceived risk of job instability on employees’ UOB through the mediating role of family financial pressure and distributive injustice. Perceived job instability threatens key resources and creates uncertainty, thereby causing stress and strain [21,22]. On the other side, job instability is depicted as a breach of the psychological contract for permanent employees [23] or as a mismatch between effort and reward in theories of social exchange (feeling job instability lessens rewards that employees obtain from their loyalty and investments) [24]. As a result, job instability reduces people’s perception of justice, which may motivate them to act unethically in order to maintain their employment [3]. Although job instability has garnered much attention, further research is necessary to better understand how employees adapt to and cope with it [25,26].

Questionnaires were designed and directed to 830 employees working in hotels (5-star and 4-star) and travel agencies (Category A). Several data analysis techniques were employed to test the structural and measurement mode. SEM was employed to test the structural model, where CFA was conducted to test the measurement model convergence and discriminant validity. The results indicated that the measurement model has good convergence and discriminant validity, and the proposed structural model fit the data well. Five hypotheses were proposed and tested. The results indicated that the perceived risk of job insecurity directly impact unethical organization behaviour and indirectly through family financial pressure and distributive injustice. The indirect effect raises the total effect of perceived risk of job instability on unethical organization behaviour by 25%, thus giving evidence that supports the partial mediation of both family financial pressure and distributive injustice on the relationship between job instability and unethical organization behaviour. All endogenous variables explain 60% of the variance in UOB.

This study proposed a model that might aid academics and practitioners in better understanding how job instability may affect employees’ UOB via the mediating effect of family financial pressure and distribution injustice. Additionally, this approach may have ramifications for practitioners. Employees who engage in unethical behaviour on behalf of their employers may be doing it for personal gain, and their actions may reflect poorly on the company’s reputation. As a result, managers should abstain from sending signals that lead employees to fear job insecurity in the workplace during a pandemic.

## Figures and Tables

**Table 1:** The profile of respondents.

		*n* = 830	%
Type of organization	Hotels	120 (430 employees)	52%
	Travel agent category A	110 (400 employees)	48%
Gender	Male	540	65%
	Female	290	35%
Marital status	Married	623	75%
	Unmarried	207	25%
Age	Less than 25 years	33	4%
	25 to 44 years	664	80%
	45 to 60 years	100	12%
	More than 60 years	33	4%
Education level	Less than high school degree	83	10%
	High school degree	150	18%
	University graduate	597	72%
Years of experience	More than 5 years	600	73%
	Less than 5 years	230	27%

**Table 2:** Descriptive analysis (*n* = 830).

Abbreviation	Items	Min.	Max.	M	S. D	Skewness	Kurt-osis
Perceived risk of job instability (Wong et al., 2021)							
J_instab1	“I have concerns about the layoff.”	1	5	3.62	1.1720	−0.490	−0.606
J_instab2	“I have concerns about salary cut.”	1	5	3.62	1.185	−0.481	−0.656
J_instab3	“I have an unstable job environment.”	1	5	3.64	1.148	−0.476	−0.597
J_instab4	“I feel emotional stress from current negative news.”	1	6	3.54	1.197	−0.475	−0.543
J_instab5	“I have insufficient resources for work (e.g., offering masks).”	1	6	3.54	1.193	−0.478	−0.529
Family financial pressure (Conger et al., 1999)							
Fin_Pres1	“My family can hardly make ends meet.”	1	5	4.14	0.679	−1.215	1.862
Fin_Pres2	“My family has difficulty paying its monthly bills.”	1	5	4.13	0.688	−1.231	1.704
Fin_Pres3	“My family has little money left at the end of the month.”	1	5	4.14	0.668	−1.206	1.110
Distributive injustice (Colquitt 2001)							
Injustice1	“I feel that the outcome process does not reflect the effort I have put into my work.”	1	5	3.63	1.230	−0.472	−0.896
Injustice2	“I feel that the outcome process is inappropriate for the work I completed.”	1	5	3.58	1.181	−0.387	−0.887
Injustice3	“I feel that the outcome process does not reflect what I have contributed to the organization.”	1	5	3.66	1.163	−0.400	−0.921
Injustice4	“I feel that the outcome process is unjustified, given my performance.”	1	5	3.53	1.201	−0.398	−0.898
UOB (Umphress et al., 2010)							
Unethic_1	“If it would help my organization, I would misrepresent the truth to make my organization look good.”	1	5	3.79	1.283	−0.984	−0.098
Unethic_2	“If it would help my organization, I would exaggerate the truth about my “company’s products or services to customers and clients.”	1	5	3.71	1.290	−0.908	−0.269
Unethic_3	“If it would benefit my organization, I would withhold negative information about my company or its products from customers and clients.”	1	5	3.76	1.265	−0.963	−0.072
Unethic_4	“If my organization needed me to, I would give a good recommendation on the behalf of an incompetent employee in the hope that the person will become another organization’s problem instead of my own.”	1	5	3.75	1.275	−0.971	−0.100
Unethic_5	“If my organization needed me to, I would withhold issuing a refund to a customer or client accidentally overcharged.”	1	5	3.70	1.307	−0.888	−0.311
Unethic_6	“If needed, I would conceal information from the public that could be damaging to my organization.”	1	5	3.69	1.328	−0.894	−0.353
Unethic_7	“I would do whatever it takes to help my organization.”	1	5	3.68	1.318	−0.867	−0.385

**Table 3:** First order factor analysis convergent and discriminant validity.

Factors and Items	Loading	CR	AVE	MSV	1	2	3	4
1—Perceived risk of job instability (*a* = 0.965)		0.960	0.827	0.043	0.910			
J_instab1	0.954							
J_instab2	0.980							
J_instab3	0.984							
J_instab4	0.808							
J_instab5	0.803							
2—Family financial pressure (*a* = 0.947)		0.947	0.856	0.043	0.207	0.925		
Fin_Pres1	0.925							
Fin_Pres2	0.942							
Fin_Pres3	0.908							
3—Distributive injustice (*a* = 0.972)		0.973	0.899	0.003	0.014	0.045	0.948	
Injustice1	0.949							
Injustice2	0.972							
Injustice3	0.945							
Injustice4	0.925							
4—UOB (*a* = 0.978)		0.978	0.863	0.018	−0.135	−0.021	−0.056	0.929
Unethic_1	0.916							
Unethic_2	0.888							
Unethic_3	0.947							
Unethic_4	0.932							
Unethic_5	0.970							
Unethic_6	0.966							
Unethic_7	0.879							

CR: composite reliability; AVE: average variance extracted; MSV: maximum shared value; diagonal values: the square root of AVE for each dimension; below diagonal values: intercorrelation between dimensions.

**Table 4:** The structural model results.

	Hypotheses			Beta (β)	C-R (*t*-Value)	R^2^	Results of Hypotheses
H1	Perceived risk of job instability	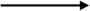	UOB	0.29 ***	5.421		Supported
H2	Perceived risk of job insecurity	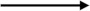	Family financial pressure	0.41 ***	9.986		Supported
H3	Family financial pressure	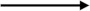	UOB	0.51 ***	13.645		Supported
H4	Perceived risk of job insecurity	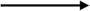	Distributive injustice	0.39 ***	7.879		Supported
H5	Distributive injustice	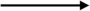	UOB	0.49 ***	12.601		Supported
UOB						0.60	

Model fit: (χ^2^ (147, *n* = 830) = 591.07, *p* < 0.001, normed χ^2^ = 4.021, RMSEA = 0.022, SRMR = 0321, CFI = 0.916, TLI = 0.986, NFI = 0.912, PCFI = 0.701, and PNFI = 0.698).; *** significance < 0.001.

## Data Availability

Data are available upon request from researchers who meet the eligibility criteria. Kindly contact the first author privately through e-mail.
